# Analysis and compensation for errors in electrical impedance tomography images and ventilation-related measures due to serial data collection

**DOI:** 10.1007/s10877-016-9920-y

**Published:** 2016-08-17

**Authors:** Rebecca J. Yerworth, Inéz Frerichs, Richard Bayford

**Affiliations:** 1 Medical Physics and Biomedical Engineering Department, University College London, London, WC1E 6BT England, UK; 2 Department of Anaesthesiology and Intensive Care Medicine, University Medical Centre Schleswig–Holstein, Campus Kiel, Kiel, Germany; 3 Department of Natural Sciences, Middlesex University, London, England, UK

**Keywords:** Lung, EIT, Imaging, Ventilation distribution, Mechanical ventilation

## Abstract

Electrical impedance tomography (EIT) is increasingly being used as a bedside tool for monitoring regional lung ventilation. However, most clinical systems use serial data collection which, if uncorrected, results in image distortion, particularly at high breathing rates. The objective of this study was to determine the extent to which this affects derived parameters. Raw EIT data were acquired with the GOE-MF II EIT device (CareFusion, Höchberg, Germany) at a scan rate of 13 images/s during both spontaneous breathing and mechanical ventilation. Boundary data for periods of undisturbed tidal breathing were corrected for serial data collection errors using a Fourier based algorithm. Images were reconstructed for both the corrected and original data using the GREIT algorithm, and parameters describing the filling characteristics of the right and left lung derived on a breath by breath basis. Values from the original and corrected data were compared using paired t-tests. Of the 33 data sets, 23 showed significant differences in filling index for at least one region, 11 had significant differences in calculated tidal impedance change and 12 had significantly different filling fractions (*p* = 0.05). We conclude that serial collection errors should be corrected before image reconstruction to avoid clinically misleading results.

**Electronic supplementary material:**

The online version of this article (doi:10.1007/s10877-016-9920-y) contains supplementary material, which is available to authorized users.

## Introduction

Electrical Impedance Tomography (EIT) images regional internal impedance changes related to physiological function using a series of surface electrodes measurement. It can achieve continuous, real-time, non-invasive, bedside monitoring of lung ventilation [1, 2]. In recent years there has been a surge of interest in its potential for monitoring regional lung ventilation [3, 4], and in particular in its application to management of acute respiratory distress syndrome in infants and adults (IRDS, ARDS). EIT has the potential of becoming a tool for optimizing ventilator therapy leading to reduced incidence of ventilator-induced lung injury. Commercial CE compliant systems are available for clinical use (www.Swisstom.com, www.draeger.com, http://www.timpel.com.br), but these are not currently configured for use with neonates.

The majority of EIT systems in clinical usage are functionally similar to the original Sheffield system [5] which collects data sequentially from different electrode combinations, a configuration with many practical advantages. However, it is assumed, during image reconstruction, that the physiological signal is quasi-static for the duration of each frame. This is often not the case. If we consider a typical system, with 16 electrodes, operating at 13 frames per second, the following consequences arise: for a neonate with a breathing rate of up to 60 breaths per minute and an even faster heart rate of 80–150 beats per minute, physiological changes will occur during the time it takes to collect one frame of data.^1^ It has been demonstrated in a single case report [6] that this introduces error of up to 4 % in the reconstructed images, and that these errors are not uniformly distributed. It was calculated that a frame rate 50 times more than the frequency of interest would be needed to reduce this effect to less than the smallest difference the system could measure [6]. Alternatively, due to the systematic nature of the errors, they can be reduced using a mathematical correction [6]. Such a correction would enable the continued use of existing data collection hardware, and an improvement in the accuracy of existing data sets. It should be noted that the ‘frequency of interest’ may be higher than the normal repetition rate of the signal if it is not sinusoidal, e.g. during inspiration there may be rapid filling followed by a slower filling phase.

This paper presents a detailed study of the effect of this error on clinical parameters derived from EIT images and the implementation of this mathematical correction method as a standalone tool. It also defines the minimum specification required for future EIT systems to monitor infants and children’s lung function.

## Data correction

Three approaches have been proposed: linear interpolation, phase correction in the frequency domain (both on the boundary voltage data) [6], and use of a regularization prior within the reconstruction algorithm which takes into account the temporal and spatial effects in the sensitivity matrix used [7]. The intention of this work was to create a standalone tool, which researchers and clinicians could use with their existing data collection, image reconstruction and analysis software.

The use of a regularization prior approach did not meet this criterion as it would require changes to the image reconstruction software used. Therefore no attempt has been made to verify the accuracy of such an approach. The frequency domain phase correction is computationally more expensive than linear interpolation, but modelling shows it to be more accurate, particularly for those EIT applications most likely to benefit from this correction (data from neonates and animal models of neonates, collected using older EIT systems, with frame rates of 10–20 frames per second).^2^ The signal from a single electrode combination was modelled as a 1 Hz sine wave, representative of neonatal breathing. This was sampled twice at 13 Hz (data1 and data2) with the sampling for data2 starting 1/26th of a second after that for data1. data1 represents the data values at the start of the data frame (the ‘true’ or ‘target’ values), and data2 represents the data values which would be measured by this representative electrode combination ~ half way through the data collection sequence. Linear and phase corrections were then applied to data2 to correct for this 1/2 frame delay, thus aligning the datasets. The point by point percentage difference between data1 and data2 was then calculated for each case.

**Fig. 1:**
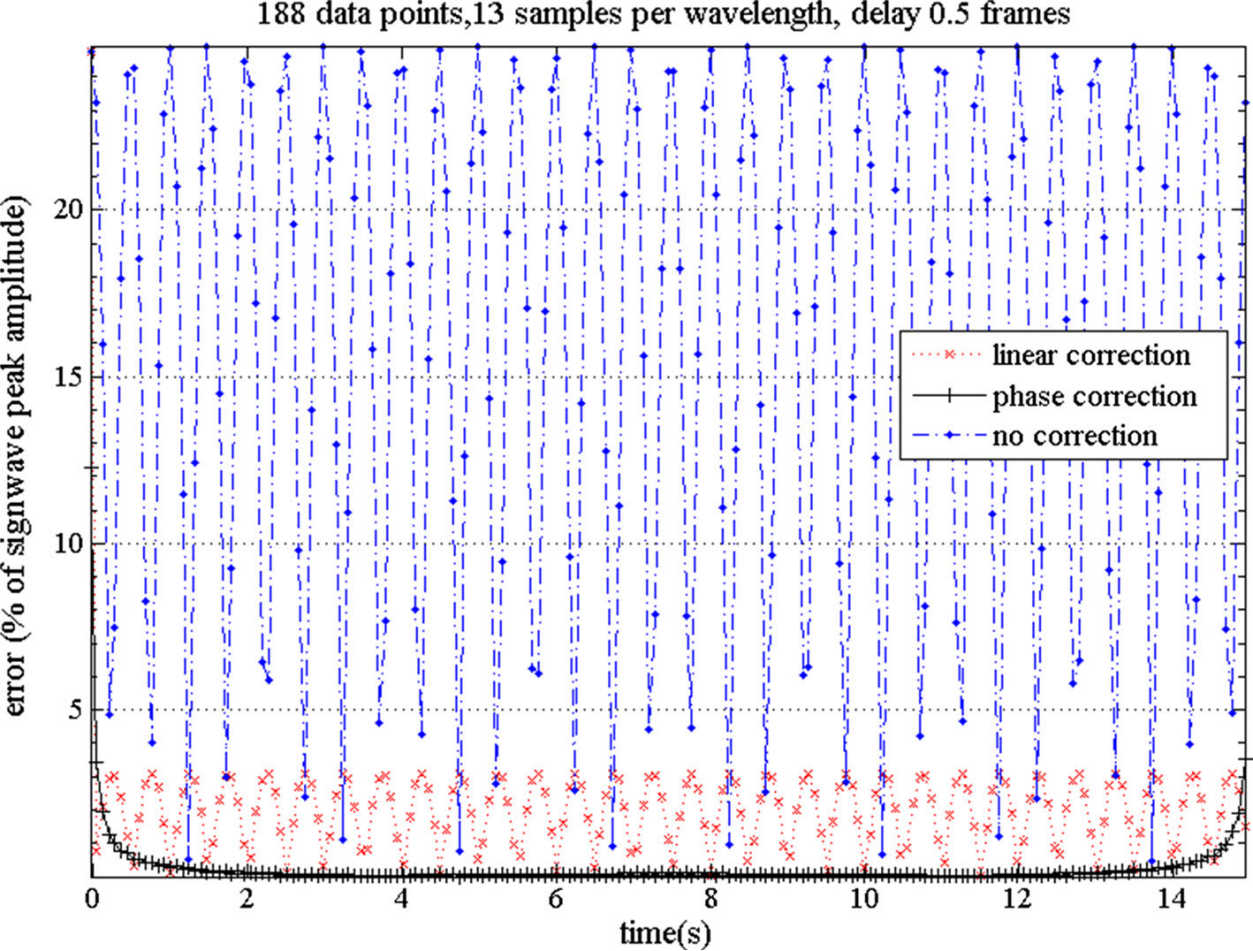
Residual error after serial data collecting for perfect sine wave representative of neonatal breathing frequency (~60 breaths per minute), EIT frame rate 13/s, assuming a collection delay of 0.5 frames. Without correction errors of up to 25 % are present (*closed circles*, *on dotted line*), with linear correction errors of up to 4 % remain (*crosses on fine dotted line*), with phase correction, excluding the 1st and last second, errors are <0.5 % (*pluses on solid line*)

Based on this modelling it can be seen that without correction, errors of up to 25 % are present (Fig. 1), with linear correction, errors of up to 4 % remain, with phase correction, excluding the first and last second, errors are less than 0.5 %. Errors after phase correction are inversely related to the sample length, so data correction should be applied to complete data files, not individual breaths. The model was also evaluated with a range of sine wave frequencies from 0.5–6 Hz, and it was found that the frequency domain phase correction consistently produced the least residue error, with the most pronounced differences at higher frequencies. This indicates that the phase correction method gives the optimum reduction in the serial data collection error, and is the method used in this paper.

## Reciprocity

To assess the quality of the data a reciprocity check is applied as part of the data correction software. Reciprocity theory states that if current is injected on electrodes a and b and measured on electrodes c and d the same results should be obtained as if measuring on a and b whist injecting on c and d [8]. However, this may be violated if there are non-idealities in the system, e.g. electrode contact impedances not perfectly matched. Changes in the physiological signal between the two measurements could also contribute to reciprocity errors. Therefore, since many EIT systems collect full data sets including reciprocity information, this theorem can be used to provide a check on the quality of data recordings, before images are reconstructed, and provide an indication of the error reduction achieved by the correction for serial data collection [6].

There is only limited research on the effect of reciprocity errors on the quality of reconstructed images. Hartinger et al. [8] demonstrated that a ‘quadrature reciprocity error’ of 0.19 (corresponding to a ~44 % reciprocity error) adversely affected the quality of reconstructed images. No smaller errors were considered. At the lower limit, reciprocity errors of <5 % can be obtained from human subjects. However, several of the data sets used to produce images for journal articles, and latter contributed to a public repository (http://eidors3d.sourceforge.net/), have large reciprocity errors on a few electrode combinations. We can conclude from this that, until further research into this is undertaken, reciprocity errors should be minimized as much as possible, preferably to <5 %.

## Filling index (FI)

**Fig. 2:**
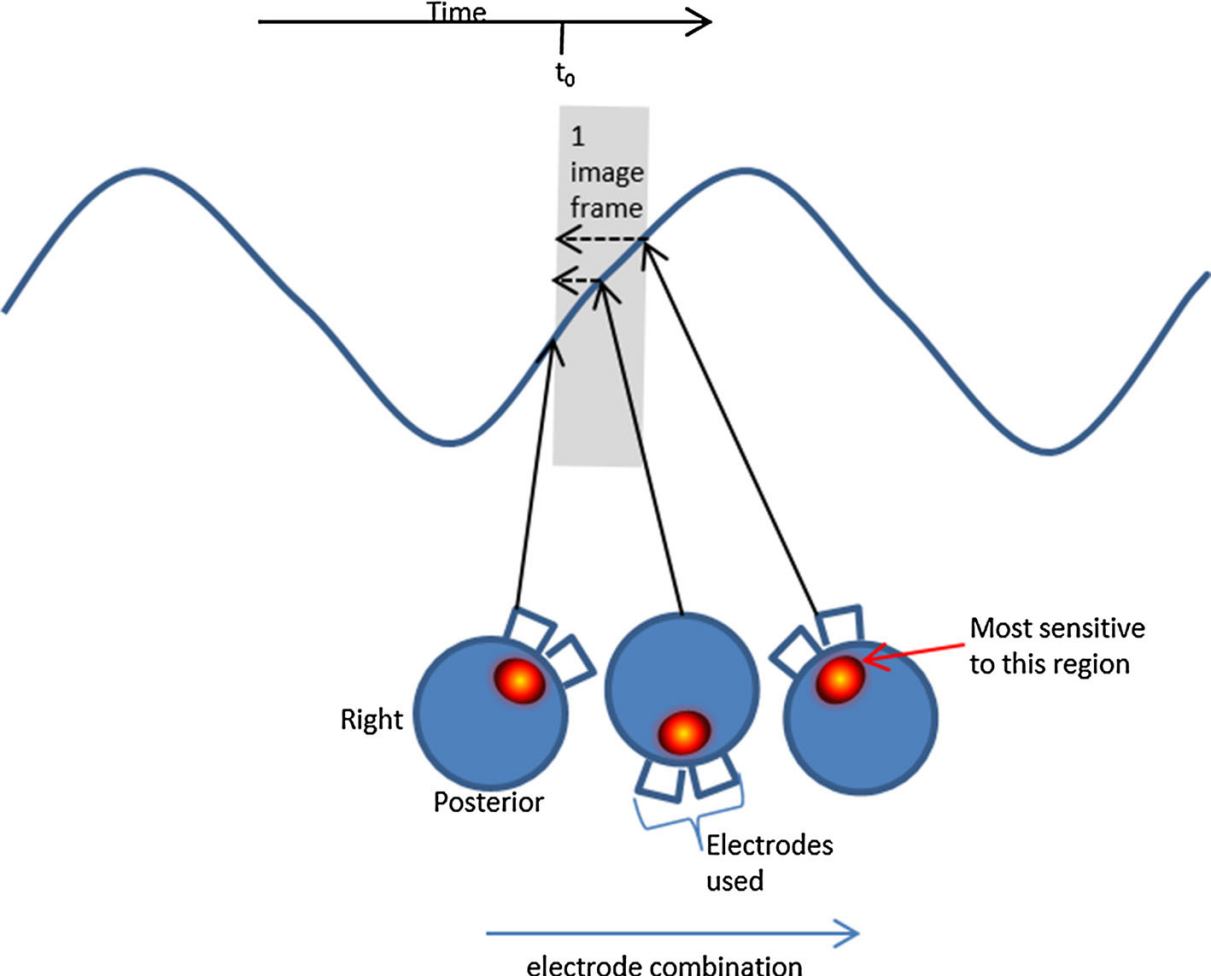
The *blue* waveform depicts the changes in lung volume occurring during ventilation. The *shaded rectangle* represents the duration of one frame of data collection. During image reconstruction, all this data is traditionally assumed to relate to the start of that data collection period (t_0_) but most is measured later, after the physiological signal has changed. During the frame, the current application rotates around the chest, shown here schematically as *three blue circles*, at three different time points, and the voltages measured are most sensitive to the properties of the nearby tissue (depicted as *red ovals*). Hence, after image reconstruction, it appears that the lung volume waveform over the right anterior region leads that of the *left anterior region*

FI indicates whether a lung region fills faster or slower than others [9]. It is calculated from the regional variations in shape of the inspiratory waveform, (Eq. 1). If all lung regions behaved the same (homogeneous) they would all have the value ‘1’ for this index. If the value is <1, that portion of lung filled faster than the others, which could be symptomatic of hyperinflation. If >1 the region filled relatively slowly, which could indicate tidal recruitment [9]. Both of which clinicians wish to avoid. The effect of serial data collection is theorized to mimic these changes: using adjacent drive and measure, all measurements for one drive combination are recorded before moving on to the next, and if electrodes are placed anticlockwise starting on the sternum, the final measurements are most sensitive to the right anterior region (Fig. 2).

The data relating to the right anterior region is collected at a later time in the breathing cycle than that to which it is attributed, and would thus appear to indicate a faster filling (FI < 1), i.e. lead the global average filling curve. Conversely the first data measurements in each frame are particularly sensitive to the left anterior quadrant, and so this region would appear to lag behind the global average filling curve (FI > 1).

## Tidal impedance change (ΔZ)

In common with papers previously mentioned [2, 4], tidal ΔZ is the magnitude of the impedance change between end-expiration and end-inspiration, either global (ΔZ_T_) or for a specific region-of-interest (ROI), e.g. the Left Anterior ROI (ΔZ_LA_).

## Filling fraction (FF)

Various measures of the relative tidal impedance change of one region relative to another are found in the literature. ‘Fractional ventilation’ is used by Heinrich et al. [10], who divided the thorax in two lung regions. Others, including Frerichs et al. [11] and Tingay et al. [12] used 64 lung regions (32 slices in each hemi-thorax) and used the phrase ‘fractional ventilation’ to represent the equivalent parameter. Regional change in tidal ventilation within each quadrant, calculated from the tidal impedance amplitude and expressed as a percentage of the summed global impedance amplitude for each recording has also been used [13] to compare the ventilation within each quadrant to the global value, and there are several variations of this [9, 14, 15]. These methods all use the same principle, but vary according to the number of regions considered and whether the answer is expressed as a percentage.

## Method

### Data

EIT boundary voltage data sets were retrospectively analysed for this study. The first was obtained from 19 spontaneously breathing neonates (mean age 30 days (range: 5–128 days), mean body weight 3029 g (range: 2150–4300 g)), treated in a neonatal ICU. Four of the neonates were also examined during the application of mechanical ventilation. In addition, data was obtained from clinical trials and experimental studies performed on a spontaneously breathing neonate, an adult human subject (age 31 years, body weight 82 kg) [16] and mechanically ventilated pigs [18, 19]. This data is on a public domain repository known as EIDORS (‘Electrical Impedance and Optical Tomography Reconstruction Software’, eidors3d.sorceforge.net).

Measurements were collected using a commercial EIT system (Goe-MF II EIT system, CareFusion, Hoechberg, Germany) with 16 electrodes placed in a single plane giving 208 electrode combinations per data frame using an adjacent drive protocol. Sections of data between 3 and 60 breaths duration of steady tidal breathing or equivalent were selected, i.e. the longest continuous section available for each subject. This variability is a consequence of the highly irregular breathing pattern in neonates, with sighs and apnoeic phases.

### Lag correction

The EIT boundary voltage measurements were processed as follows, using software written in Matlab [www.mathsworks.com]: for each image frame (208 voltage measurements), a Fast Fourier Transform (FFT) was applied, then correction for lag was made by applying a frequency dependent phase adjustment and considering the relative delay on the nth data point to be (n–1)/208th of the frame rate, before doing an inverse FFT [6].

The percentage of electrode combinations with a median absolute reciprocity of >5 % was calculated using the lag corrected data. Additionally, the absolute percentage difference between original and corrected data as mean (±std) of all electrode combinations was calculated.

### Image reconstruction

For each original and corrected data set separately, time points at peak inspiration and expiration were determined. The data from all electrode combinations was averaged and low-pass filtered to remove heart-beat related effects. The stationary points were identified. These were considered ‘end-inspiration’ if the impedance at that time was greater than the mean, and the immediately preceding minima was deemed ‘end-expiration’. The points were reviewed for accuracy of breath detection, and regions of tidal breathing manually identified.^3^ Images were reconstructed using the GREIT algorithm [17] with the included neonate or pig mesh, as appropriate, and using the mean of all selected breaths as the reference. The resultant images were rasterised on to a rectangular grid. The “*Breathing periodicity*” was calculated as the mean number of data frames between sequential end expiration times- that is the data collection rate normalized to the breathing rate.

### Image parameters

**Fig. 3:**
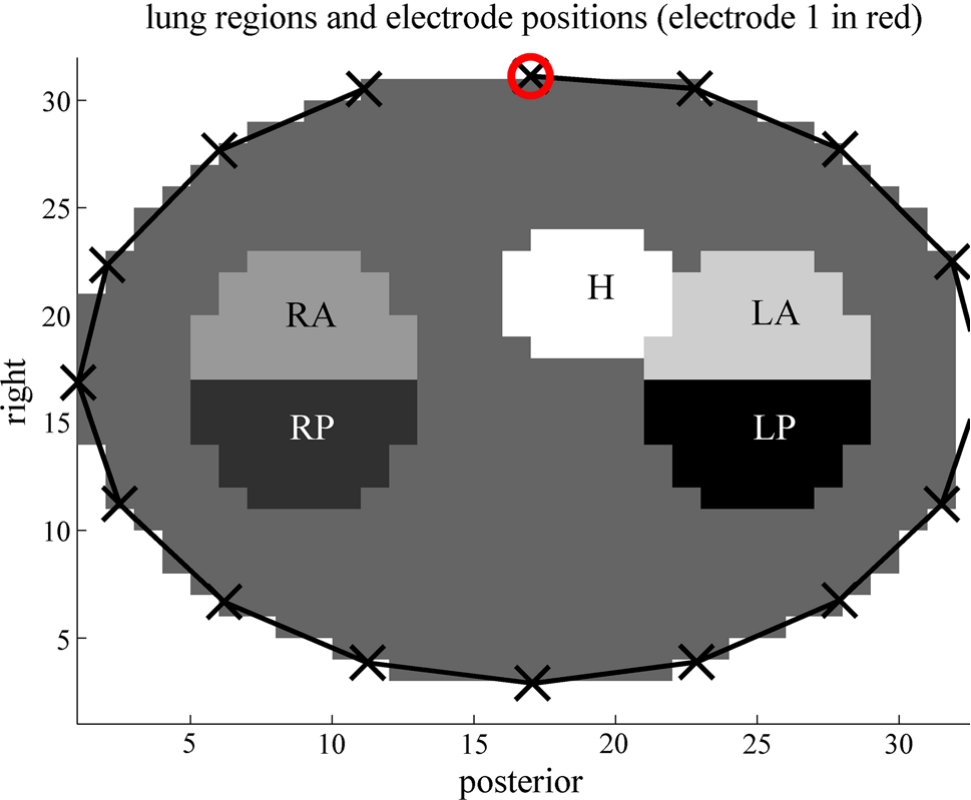
Regions in neonate mesh (4 lung *quadrants*: left anterior (*LA*), right anterior (*RA*), left posterior (*LP*) and right posterior (*RP*)), the heart (*H*) and the positions of the first electrode (o) on the anterior *midline*, and the other electrodes (x) clockwise from this, evenly spaced around the surface (32 × 32 pixels)

Posterior and anterior regions of each lung were defined: left anterior (LA), right anterior (RA), left posterior (LP) and right posterior (RP) (Fig. 3). For each data frame, for images reconstructed from the original and corrected data separately, the sum of the pixel values (relative impedance, Z) of each lung quadrant was determined. The following parameters were then calculated, on a breath by breath basis for each of the 4 lung quadrants:

1. *Filling index (FI):* the inspiratory phase of each breath was fitted to the model of form:

1 $$Z\left( {Z_{g} } \right) = a \times Z_{g}^{FI} + c$$

The vector Z is the relative impedance of the relevant lung quadrant from each image during inspiration and is assumed to be a function of vector Z_g_, the average of the 4 quadrants. FI, a and c are constants, derived during the fitting.

(2) Δ*Z:* Change in relative impedance between end-expiration and end-inspiration (arbitrary units).
(3) *Filling fraction (FF):* For each breath regional FF = ΔZ/ΔZ_T_.

Where ΔZ_T_ is the summed ΔZ of the 4 quadrants

Paired t-tests were conducted between the original and corrected results for each ROI, for each subject. Null hypothesis were of the form:

#### **H0**_**1**_

 Mean (ΔZ_RL_ (original) − ΔZ_RL_ (corrected)) = 0 for subject 1.

## Results

Of the 19 spontaneously breathing infants analysed (Online resource 1: Table 1) 12 showed significant changes in FI within at least one quadrant, 6 had significant changes in ΔZ and 7 significant changes in FF. The mean breathing periodicity was 18.5 ± 6.0 frames per breath, 49 ± 11 % of inspiratory breathing flags were shifted 1 data collection frame later by data correction, and 55 ± 13 % of expiratory flags. In all cases lag correction significantly reduced the frame by frame reciprocity error. Four infants (numbers 15–18) who were also mechanically ventilated (Online resource 1: Table 2), all showed a very highly significant increase in FI within the left anterior region, and 3 very significant decreases in FI within the right anterior and right posterior regions. Decreases were also seen in the 4^th^ data set, but these were not significant. One data set also showed significant decreases in ΔZ and FF within the left anterior region. The mean breathing periodicity was 32.9 ± 5.2 frames per breath, 51 ± 8 % of inspiratory breathing flags were shifted 1 data collection frame latter by data correction, and 58 ± 15 % of expiratory flags. In all cases lag correction significantly reduced the frame by frame reciprocity error. Ten other data sets were analysed, and similar results were found (Online resource 1: Table 3).

**Fig. 4:**
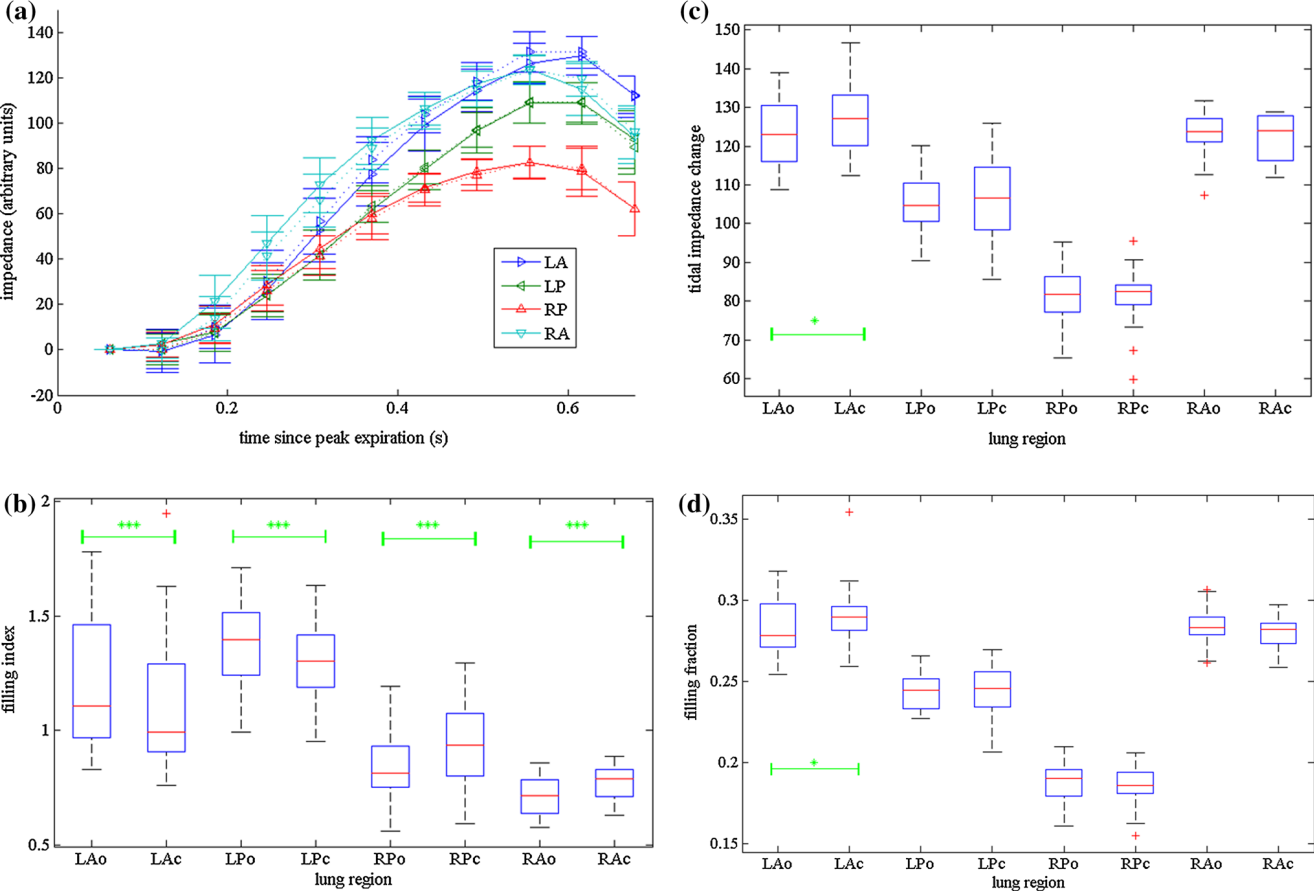
Analysis of EIT data acquired in a 92-d old mechanically ventilated neonate with a body weight of 3800 g. The baby was prematurely born (gestational age: 33 weeks, body weight at birth 1960 g) **a** original (*solid lines*) and corrected (*dashed lines*) impedances curves, all breaths overlaid, **b** boxplot of Filling Index (*FI*) **c** boxplot of tidal impedance change (ΔZ) **d** box plot of the Filling Fraction (FF). *Stars* denote significance of difference between corrected and uncorrected data: *p < 0.5, **p < 0.01, ***p < 0.001. *LA* left anterior, *RA* right anterior, *LP* left posterior, *RP* right posterior. *Suffix ‘o’* Original data, Suffix ‘c’ corrected data

**Fig. 5:**
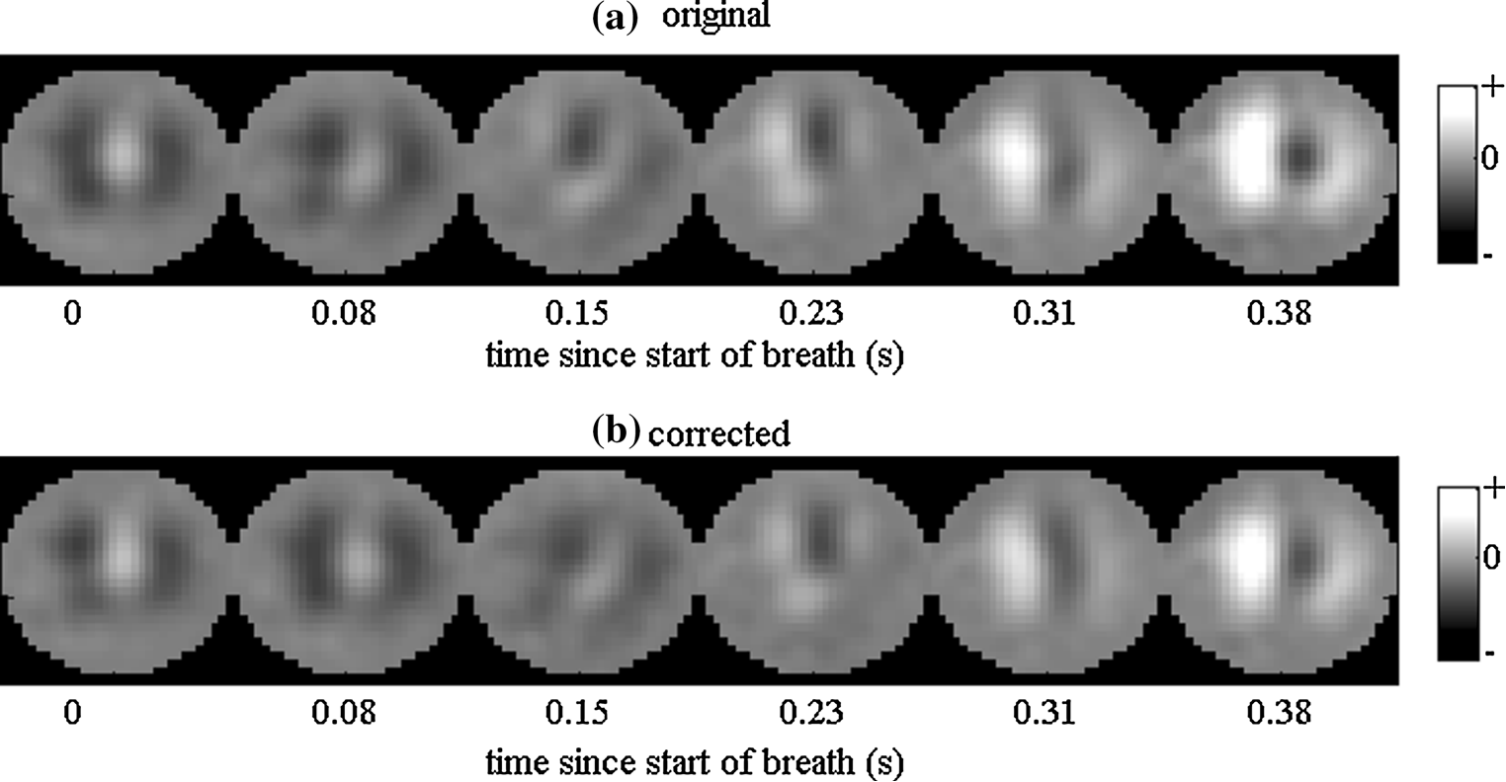
Original and corrected images of the same neonate as in Fig. 4, corresponding to the last inspiration within the analyzed time period, showing the apparently quicker filling of the right lung being reduced by the correction (*white* denotes increased impedance relative to baseline, arbitrary units, and is indicative of air entering lung): For both sets of images, the extent and intensity of the increase is greater over the right lung region than the left, from 0.23 s. However, the difference is less marked when the corrected data is reconstructed

Figure 4 illustrates the results for one of the mechanically ventilated neonates, number 16 in Online resource 1: Table 2. This shows; (a) the mean regional impedance as a function of time for the original and corrected data (inspiratory section only), (b–d) a boxplot of each parameter in turn: (b) FI, (c) ΔZ and (d) FF values. For each region, the FI of the corrected data shows a significant difference from that for the original data and is also nearer to 1, indicating less spatial inhomogeneity in the corrected data. Both right lung regions have a less negative FI, whilst both left lung regions show a less positive FI. Reconstructed images for the first 6 frames indicate earlier impedance changes in the right lung when using the original data than with the corrected data (Fig. 5). A colour version of this figure is included as supplementary material online (Online resource 2). For this data set, ΔZ and FF are significantly different in the left anterior region only. The other data sets show the same trends, with significant changes in FI in at least one region for 23 of the 33 data sets. Eleven showed significant changes in ΔZ and 12 had significant changes in FF.

## Discussion

**Fig. 6:**
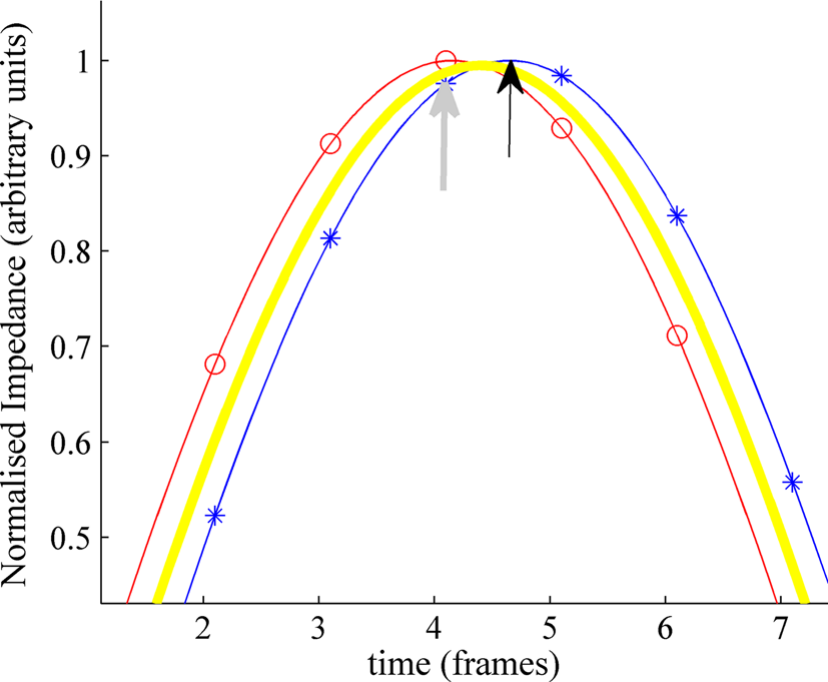
Understanding the effect on ΔZ, part of a sine wave representative of the end-inspiration section of one breath, without serial error correction. *Solid line* with *open circles* right lung; *solid line* with *stars* left lung; *thick line* global average. *Black arrow* true maximum impedance of left lung; *grey arrow* reported maximum impedance of left lung

The results presented in this paper confirm that systems that measure data serially can significantly alter the interpretation of reconstructed EIT images if the frame rate per image is not sufficient to capture the change in physiology. In all the data analysed in this paper end-expiration and end-inspiration were found to be shifted for at least 33 %, and typically more than half of the breaths. The mechanism associated with the shift, and its variability, can be explained as follows: consider two sine waves of identical frequency and amplitude, that represent the volumetric changes in the left and right lungs respectively, but with a phase difference equivalent to one wave being recorded with a delay equivalent to 0.5 of the frame duration (Fig. 6). After correction, the right lung will have the same phase as the left (thin line with stars). However, before correction, end-inspiration (maximum amplitude of sine wave) is calculated from the global average (i.e. average of the two signals, thick line) and occurs earlier than it does in the corrected data. To complicate things further, breathing times are rounded to the nearest frame generating an additional timing error of up to ±0.5 of a frame in both cases. Thus, the original and corrected data end-inspiration times will sometimes synchronise, and sometimes be one frame apart, depending on the instantaneous phase relationship between the physiological signal and data acquisition.

This has implications for other parameters including ΔZ. All measurements will be subject to a variable error due to the measurements not being synchronised with end-inspiration and end-expiration. For example, if there are 15.7 frames per breath (Fig. 6), errors of up to ~2 % could be present, with the errors always being a reduction. The slower the data acquisition, relative to the breathing rate, the higher this error becomes (e.g. if 4 frames per breath, the worst case scenario is when the data points are at π/4, 2π/4 etc., which gives a 15 % error ((1–sin(π/4))/2) rising to 100 % at the Nyquist limit).

In addition to this we have the serial collection effect. At the time when the mean global impedance change is maximum (frame 4.4, Fig. 6), data relating to the right lung is from the early expiration phase, whereas data from the left lung will be from late inspiration, so both ΔZ s will be less than the true value. Theoretically, the true ΔZ will be obtained after correction. However, if the peaks do not exactly coincide with the start of a data acquisition frame, the rounding effect will remain. So on average, ΔZ would increase following correction. However, the subtle interactions between serial collection effects and peak/data acquisition synchrony, combined with the non-sinusoidal shape of the breathing cycle, will cause these errors to partially counteract each other, and ΔZ may actually be lower for a particular quadrant. This is present in the results: in more than 60 % of cases ΔZ corrected was greater than ΔZ original (resulting in a negative difference), and the average difference was negative for all quadrants. For one of the spontaneously breathing neonates (age: 43 days, body weight: 3185 g) the median ΔZ changed from 79 to 88 (arbitrary units) and the mean change was –6.3 (original—corrected) this is ~8 % change.

FI is the derived parameter most susceptible to errors due to serial collection timing errors. This is unsurprising as it is explicitly dependent on the timing of the most rapidly changing phase of the breathing cycle. Changes of 0.1–0.2 have been demonstrated in most of the data sets. These are of similar magnitude and direction to the differences between left and right lung regions reported in rats [9], and in adult humans [18]. Thus serial correction errors could either mask or be misinterpreted as physiologically significant effects. To prevent this, serial error correction must be applied before clinical diagnoses can be made.

Significant changes in FF were seen in 40 % of the data sets. Positive and negative changes were seen and there is no significant relationship between polarity and region. As discussed earlier there are two interplaying errors in the original data -serial collection related and rounding to the nearest frame. After correction only the rounding error remains and should largely cancel out, e.g. if the time of end-inspiration is rounded down then all the data will be slightly less than the peak by the same fraction of peak intensity, unless the time course of the impedance change in each region is significantly different, i.e. FI ≠ ~1. However, if before correction the end-inspiration time was rounded down, the left-hand lung regions would have the largest errors, whereas if rounded up the right-hand regions would be the most inaccurate, thus the direction of the original rounding determines the polarity of the FF changes. The same holds true for end-expiration, and these errors are additive.

The data was inspected to look for predictors of significant changes in the parameters (FI, ∆Z & FF). Whilst no clear trends were found, the highest contributing factors are likely to be breathing periodicity and number of breaths averaged; there are hints of this in the results, but a much larger sample size, with tighter inclusion criteria, would be needed to confirm this.

Although the reconstruction algorithms may vary between different serial data collection systems, these results and the underpinning theory are universally applicable. Mathematical modelling shows that this serial error can only be safely ignored for data collection rates of more than 50 frames per breath (or 50 frames per cardiac cycle, if that signal is large or of interest) [6].

The correction tool^4^ used for this paper is tailored for use with *.get files (Goe-MF II EIT system, CareFusion, Hoechberg, Germany) obtained with the standard 16 electrode, 208 measurements per frame protocol with any frame rate and number of frames. However, it could be modified for use with any serially collected EIT data. The software also should be used, where possible, to check the quality of data with respect to reciprocity before starting the main data collection runs. Further work is needed to establish the limit at which reciprocity errors significantly affect reconstructed images. In the meantime a conservative threshold for reciprocity error warnings has been set, which may be un-necessarily stringent.

## Conclusions

The authors recommend that users of serial EIT systems apply this serial correction algorithm to their data before analysis, or use a frame rate of greater than 50 frames per breath and consider re-analysing previously collected data allowing more accurate information to be obtained.

## Electronic supplementary material

Below is the link to the electronic supplementary material.

Supplementary material 1 (PDF 246 kb)

Supplementary material 2 (PDF 351 kb)

## Footnotes

1 For a breathing rate of 60 breaths per minute, i.e. 1 breath per second, if inspiration and expiration phases are equal there are 6–7 data points during the inspiratory phase and 6–7 during the expiratory. This corresponds to a change of >1/6th (17 %) of the inspired volume occurring during a single frame of data collection. However the inspiratory phase is often shorter than the expiratory, so this is an underestimate of the physiological changes present.

2 Most recently developed commercial EIT devices do not offer specific electrode interfaces for neonates, therefore these older EIT devices are the only ones available for this patient population.

3 A more sophisticated breath detection approach would be needed for unsupervised use and non-tidal breathing.

4 Available from EIDORS http://eidors3d.sourceforge.net/ or by contacting the authors..
