# Supplementary material for: Analysis and compensation for errors in electrical impedance tomography images and ventilation-related measures due to serial data collection
Source: J Clin Monit Comput. 2016 Aug 17;31(5):1093–101. doi: 10.1007/s10877-016-9920-y (PMC5599443; doi:10.1007/s10877-016-9920-y)
Supplement: Supplementary file 1 — Supplementary material 1 (PDF 246 kb) [file 10877_2016_9920_MOESM1_ESM.pdf]

Online material 2

Color version of Figures in:

Analysis and compensation for errors in electrical impedance tomography images and ventilation-related measures due to serial data collection.

Submitted to Journal of Clinical Monitoring and Computing (JCMC)

Authors: Rebecca J. Yerworth, Inéz Frerichs, and Richard Bayford

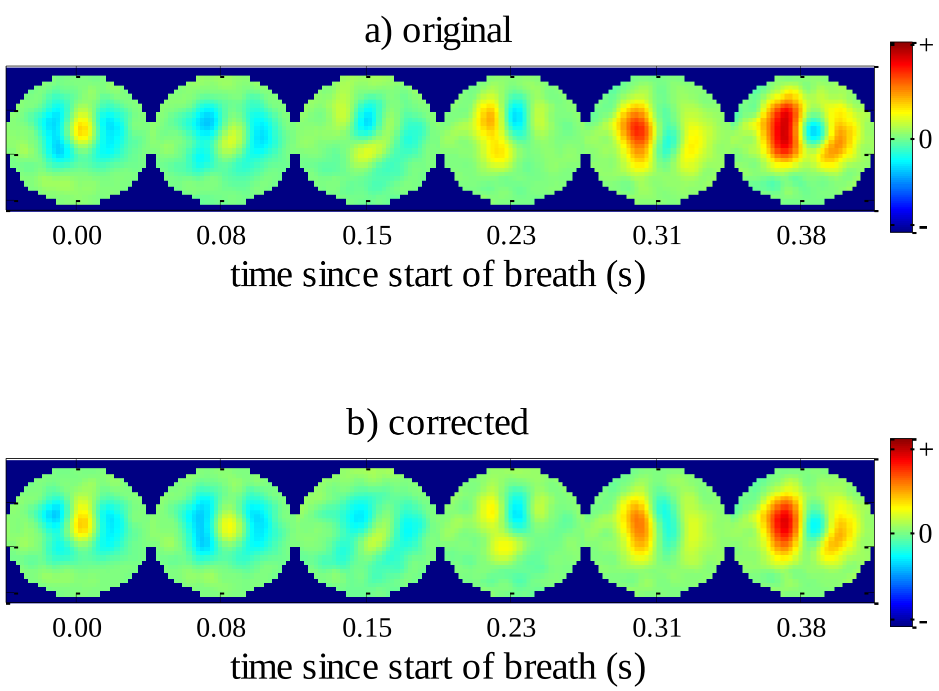

Fig. 5 Original and corrected images of the same neonate as in Fig. 4, corresponding to the last inspiration within the analyzed time period showing the apparently quicker filling of the right lung being reduced by the (red denotes increased impedance relative to baseline, arbitrary units, and is indicative of air entering lung): For both the sets of images, the extent and intensity of the increase is greater over the right lung region than the left, from 0.23s. However the difference is less marked when the corrected data is reconstructed.
