# Supplementary material for: Analysis and compensation for errors in electrical impedance tomography images and ventilation-related measures due to serial data collection
Source: J Clin Monit Comput. 2016 Aug 17;31(5):1093–101. doi: 10.1007/s10877-016-9920-y (PMC5599443; doi:10.1007/s10877-016-9920-y)
Supplement: Supplementary file 2 — Supplementary material 2 (PDF 351 kb) [file 10877_2016_9920_MOESM2_ESM.pdf]

Online material 1

Tables relating to:

Analysis and compensation for errors in electrical impedance tomography images and ventilation-related measures due to serial data collection.

Submitted to Journal of Clinical Monitoring and Computing (JCMC)

Authors: Rebecca J. Yerworth, Inéz Frerichs, and Richard Bayford

TABLE 1

SPONTANEOUSLY BREATHING NEONATES

	FI: mean diff				ΔZ: mean diff (arbitrary units)				FF:mean diff				no.
	LA	LP	RP	RA	LA	LP	RP	RA	LA	LP	RP	RA	breaths
1	0.163	0.060	-0.105	-0.160	-2.702	4.248	-0.085	-2.714	-0.005	0.009	0.001	-0.006	7
2	0.109	0.066	-0.055	-0.072	-1.271	-1.198	-1.591	-6.341	0.007	0.003	0.002	-0.012	47
3	0.027	-0.024	0.006	-0.067	-3.557	-6.267	-13.338	-8.841	0.010	0.002	-0.009	-0.004	10
4	0.262	0.040	-0.091	-0.177	1.201	0.661	-2.191	-1.086	0.007	0.005	-0.008	-0.004	23
5	0.082	0.028	-0.010	-0.125	-4.331	-3.091	-15.099	-11.692	0.007	0.012	-0.008	-0.011	8
6	0.004	0.095	-0.007	0.000	-3.236	1.839	-2.308	3.279	-0.008	0.004	-0.005	0.009	5
7	0.123	0.067	-0.030	-0.150	-0.179	-1.007	0.017	-0.780	0.001	0.000	0.000	-0.001	11
8	0.174	0.053	-0.061	-0.027	-5.202	-0.763	-3.934	-4.032	-0.008	0.009	0.004	-0.004	26
9	-0.017	0.029	-0.013	-0.097	-2.492	4.170	0.836	0.742	-0.010	0.009	0.000	0.001	16
10	0.238	-0.192	0.185	-0.217	-6.131	2.164	-8.091	1.988	-0.008	0.017	-0.018	0.009	13
11	0.125	0.0264	-0.027	-0.193	-0.653	-0.918	-1.541	-0.657	0.001	0.000	-0.002	0.001	26
12	0.163	0.049	-0.081	-0.057	2.083	-1.205	0.553	0.257	0.005	-0.003	0.002	-0.004	7
13	-0.017	-0.001	-0.068	-0.150	-4.556	0.897	5.552	-3.177	-0.011	0.005	0.015	-0.009	5
14	0.060	0.161	-0.064	-0.196	-3.074	-2.414	-3.952	-5.610	-0.002	0.005	0.003	-0.005	15
15	0.055	0.173	-0.055	-0.149	-0.323	-3.518	-4.778	-2.611	0.005	-0.003	-0.001	-0.001	12
16	0.100	0.045	-0.047	-0.177	-8.960	3.470	5.950	1.605	-0.024	0.008	0.012	0.004	7
17	0.067	0.047	-0.120	-0.109	2.022	-2.724	0.390	0.203	0.006	-0.008	0.002	0.000	3
18	-0.191	0.078	0.026	0.018	8.599	2.014	-6.656	-5.235	0.072	0.026	-0.051	-0.047	16
19	0.208	-0.161	0.007	-0.013	12.704	7.951	0.688	5.324	0.035	-0.011	-0.039	0.015	5
mean	0.091	0.034	-0.032	-0.111	-1.056	0.227	-2.609	-2.072	0.004	0.005	-0.005	-0.004	14
stdev	0.106	0.088	0.066	0.072	5.037	3.357	5.391	4.225	0.020	0.009	0.016	0.012	10.7

Change in clinical parameters filling index (FI), impedance change (ΔZ) and filling fraction (FF) due to correction for serial data collection for 19 mechanically ventilated infants. LA: left anterior; RA: right anterior; LP: left posterior; RP: right posterior.

Light shading, black font: $p<0.05$; light shading white font: $p<0.01$; dark shading with white font: $p<0.001$.

TABLE 2

MECHANICALLY VENTILATED NEONATES

	FI: mean diff				ΔZ: mean diff (arbitrary units)				FF:mean diff				no.
	LA	LP	RP	RA	LA	LP	RP	RA	LA	LP	RP	RA	breaths
15	0.046	0.018	-0.048	-0.019	-4.627	0.917	0.179	0.038	-0.003	0.001	0.001	0.001	9
16	0.101	0.080	-0.096	-0.056	-3.477	-0.333	0.559	1.109	-0.007	0.001	0.002	0.004	24
17	0.150	0.038	-0.119	-0.074	-1.684	0.238	1.423	1.504	-0.005	0.000	0.002	0.003	15
18	0.073	0.040	-0.082	-0.038	-3.045	-0.270	1.500	0.347	-0.006	0.000	0.004	0.002	27
mean	0.09	0.04	-0.09	-0.05	-3.21	0.14	0.92	0.75	-0.005	0.000	0.002	0.002	18.75
stdev	0.04	0.03	0.03	0.02	1.22	0.58	0.65	0.67	0.002	0.001	0.001	0.001	8.26136

Change in clinical parameters filling index (FI), impedance change (ΔZ) and filling fraction (FF) due to correction for serial data collection for 4 mechanically ventilated infants. LA: left anterior; RA: right anterior; LP: left posterior; RP: right posterior.

Light shading, black font: $p<0.05$; light shading white font: $p<0.01$; dark shading with white font: $p<0.001$.

TABLE 3

OTHER DATA SETS

	FI: mean diff				ΔZ: mean diff (arbitory units)				FF:mean diff				no.
	LA	LP	RP	RA	LA	LP	RP	RA	LA	LP	RP	RA	breaths
P1	0.166	0.016	-0.035	-0.118	2.252	-3.761	-1.614	5.356	0.003	-0.007	-0.003	0.007	42
N1	0.127	0.142	-0.081	-0.167	0.276	-3.086	-1.149	-0.942	0.006	-0.006	0.000	0.000	58
N2	0.155	0.118	-0.086	-0.213	-3.777	-2.348	0.608	1.030	-0.015	-0.003	0.010	0.008	12
A1	0.026	0.020	-0.014	-0.059	-1.619	1.646	0.736	-1.103	-0.003	0.003	0.001	-0.001	11
A2	0.020	0.015	-0.012	-0.019	1.546	0.895	-0.250	0.552	0.001	0.000	-0.002	0.000	10
A3	0.021	0.015	-0.019	-0.033	0.170	-0.400	-2.153	-2.626	0.003	0.002	-0.002	-0.003	12
A4	0.021	0.011	-0.008	-0.048	-0.259	-2.413	-1.814	-2.060	0.002	0.000	0.000	-0.001	12
A5	0.015	0.042	0.011	0.019	3.596	1.924	-0.383	1.201	0.003	0.007	-0.010	0.000	13
P2	-0.055	0.003	-0.003	-0.004	-10.819	-3.713	-1.848	-9.802	-0.018	0.004	0.005	0.010	9
P3	0.131	0.099	-0.082	0.024	7.701	-0.204	-1.815	-2.672	0.030	-0.002	-0.008	-0.019	8

Change in clinical parameters filling index (FI), impedance change (ΔZ) and filling fraction (FF) due to correction for serial data collection for 3 pigs (P1 [18], P2 & P3 [19]), mechanically ventilated; one spontaneously breathing infant (N1, N2); and one spontaneously breathing adult in a range of postures (sitting (A1,A2), lying on right side (A3), supine (A4,A5)[17]). LA: left anterior; RA: right anterior; LP: left posterior; RP: right posterior.

Light shading, black font: $p<0.05$; light shading white font: $p<0.01$; dark shading with white font: $p<0.001$.
